# Differential Brain Development with Low and High IQ in Attention-Deficit/Hyperactivity Disorder

**DOI:** 10.1371/journal.pone.0035770

**Published:** 2012-04-20

**Authors:** Patrick de Zeeuw, Hugo G. Schnack, Janna van Belle, Juliette Weusten, Sarai van Dijk, Marieke Langen, Rachel M. Brouwer, Herman van Engeland, Sarah Durston

**Affiliations:** 1 Neuroimaging Lab, Department of Child and Adolescent Psychiatry, Rudolf Magnus Institute of Neuroscience, University Medical Center Utrecht, Utrecht, The Netherlands; 2 Structural Neuroimaging Group, Department of Psychiatry, Rudolf Magnus Institute of Neuroscience, University Medical Center Utrecht, Utrecht, The Netherlands; King's College London, United Kingdom

## Abstract

Attention-Deficit/Hyperactivity Disorder (ADHD) and intelligence (IQ) are both heritable phenotypes. Overlapping genetic effects have been suggested to influence both, with neuroimaging work suggesting similar overlap in terms of morphometric properties of the brain. Together, this evidence suggests that the brain changes characteristic of ADHD may vary as a function of IQ. This study investigated this hypothesis in a sample of 108 children with ADHD and 106 typically developing controls, who participated in a cross-sectional anatomical MRI study. A subgroup of 64 children also participated in a diffusion tensor imaging scan. Brain volumes, local cortical thickness and average cerebral white matter microstructure were analyzed in relation to diagnostic group and IQ. Dimensional analyses investigated possible group differences in the relationship between anatomical measures and IQ. Second, the groups were split into above and below median IQ subgroups to investigate possible differences in the trajectories of cortical development. Dimensionally, cerebral gray matter volume and cerebral white matter microstructure were positively associated with IQ for controls, but not for ADHD. In the analyses of the below and above median IQ subgroups, we found no differences from controls in cerebral gray matter volume in ADHD with below-median IQ, but a delay of cortical development in a number of regions, including prefrontal areas. Conversely, in ADHD with above-median IQ, there were significant reductions from controls in cerebral gray matter volume, but no local differences in the trajectories of cortical development.

In conclusion, the basic relationship between IQ and neuroanatomy appears to be altered in ADHD. Our results suggest that there may be multiple brain phenotypes associated with ADHD, where ADHD combined with above median IQ is characterized by small, more global reductions in brain volume that are stable over development, whereas ADHD with below median IQ is associated more with a delay of cortical development.

## Introduction

Attention-Deficit/Hyperactivity Disorder (ADHD) is associated with substantial heterogeneity in terms of its cognitive correlates, changes in brain development, and genetic influences [1]–[4]. Due to this heterogeneity, it has proven difficult to detect the etiological cascades that lead to symptoms of the disorder. One approach to address this may be to parse the phenotype along etiologically informative characteristics. General intelligence or IQ may be one such characteristic, given growing evidence that it is an important source of heterogeneity in ADHD.

ADHD and IQ are both highly heritable phenotypes, with heritability estimates between 70–80% for both [5], [6]. Furthermore, IQ has been suggested to co-segregate with ADHD in families: a sibling study showed that children with ADHD had the lowest and controls the highest IQ scores, while the siblings of probands had intermediate scores [7]. Twin studies have shown that the relationship between ADHD and IQ is almost entirely explained by shared genetic factors [8], [9]. Importantly, this does not appear to be merely an epiphenomenon of the relationship between ADHD and established cognitive endophenotypes, such as cognitive control, as the genetic factors affecting IQ are disparate from those influencing other cognitive endophenotypes in ADHD [7], [10], [11].

The relationship between ADHD and IQ is also relevant clinically: An average reduction in IQ of 9 scale points has been reported across studies [12]. This reduction appears to be attenuated in adults with ADHD and in nonclinical samples [13]. However, lower IQ has also been associated with poor treatment response [14]–[17], and has been shown to negatively affect long-term functional outcome [18], [19].

Based on these findings, it has been suggested that the shared genetic effects associated with both IQ and ADHD may be reflected in shared neuroanatomical changes [8]. Neuroanatomical differences in frontostriatal areas, parietal and anterior cingulate cortex and the cerebellum have been consistently reported in ADHD, in both structural MRI and Diffusion Tensor Imaging (DTI) studies [2], [3], [20], [21]. A large number of studies in typically developing children and adults have reported positive correlations between IQ and anatomical brain measures [22]–[41]. A meta-analysis of these studies found an unbiased correlation in the population between IQ and total brain volume of r = .33 across 37 studies of both adult and child samples [36]. More specifically, lateral prefrontal cortex, parietal association cortex and, to a lesser extent, temporal cortex appear to be particularly correlated with IQ [32], [42], [43], and the efficiency of the network between these regions has been proposed to form its functional correlate [32], [44]–[46]. A smaller number of studies have addressed the relationship between IQ and white matter integrity but despite differences in analytical approach, find similar relationships between IQ and these brain measures [35], [38], [39], [42]. Twin studies have indicated that shared genetic effects largely explain the relationship between IQ and neuroanatomical measures in adults [22], [23], [27], [31], [47], [48], and children [49]–[51]. Taken together, this evidence suggests that there may be overlap in the neuroanatomical correlates of both ADHD and IQ. As a result of this, the neuroanatomical profile of ADHD may not be constant across the IQ scale.

Cortical thickness has proven to be particularly sensitive to developmental effects in both longitudinal and cross-sectional studies [26], [52]–[54]. A series of studies by Shaw and colleagues showed that the development of cortical thickness varies across different levels of intellectual capacity [25], and that cortical development appears to be delayed by up to five years in children with ADHD compared to typically developing controls [55], [56].

Despite accumulating evidence that IQ is phenotypically important in ADHD, it is still often treated as nuisance variance or included as a covariate in analyses. However, actively investigating how differences in IQ relate to the neuroanatomical signature of disorders may be a more informative approach, in particular for ADHD, where changes in IQ are part of the clinical phenotype. Therefore, we set out to investigate the relationship of IQ and brain development in a sample of children with ADHD and typically developing controls. As overlapping genetic effects have been shown to influence both ADHD and IQ, and both are associated with brain changes, we hypothesized that IQ would act as a statistical moderator of brain differences associated with ADHD. Note that we did not test whether a difference in IQ precedes or causes brain changes in ADHD. We simply tested whether neuroanatomical differences associated with ADHD vary as a function of IQ. Specifically, we hypothesized that changes in the developmental pattern of cortical thickness would be greatest in children with ADHD and below median IQ, as ADHD is associated with a delay in cortical peak thickness [55] and the cortical peak in typical development occurs earlier with lower IQ [25], thus maximizing the likelihood of detecting a difference. Following a similar line of reasoning, we hypothesized that ADHD combined with above median IQ would be associated with widespread reductions in cortical thickness that have been reported in earlier studies on cortical thickness in ADHD [55]–[60], but following the same developmental trajectory as controls. As a result, we hypothesized that the typical correlations between measures of brain anatomy and IQ would be absent in ADHD with increasing volumetric deviation from controls across the IQ span.

## Methods

### Participants

A total of 214 children aged 6 to 15 years participated in this study. A subset of 200 children (101 control subjects, 99 subjects with ADHD, matched at the group level for age, gender and hand preference), participated in a structural MRI session. In order to assess the developmental trajectory of cortical thickness in subgroups differing in IQ, we performed a group split at the median IQ for the whole group (IQ = 102) to form four subgroups (controls with above median IQ, controls with below median IQ and children with ADHD with above median and below median IQ). There were no differences in age, gender, or hand preference between these subgroups. The below median and above median IQ groups of controls and subjects with ADHD did not differ in mean IQ. We acquired Diffusion Tensor Imaging (DTI) scans from a smaller subsample, largely overlapping with the first sample (34 controls, 30 subjects with ADHD; overlap: 29 controls, 21 subjects with ADHD). These data were used to assess the effect of IQ on overall cerebral white matter microstructure. Table 1 provides demographic information for both samples.

**Table 1:** Demographic data.

		Controls				ADHD				Tests for Group Differences		
		Anatomical MRI data			DTI data	Anatomical MRI data			DTI data	Anatomical MRI data		DTI data
		All (N = 101)	Below median IQ (N = 40)	Above median IQ (N = 61)	All (N = 34)	All (N = 99)	Below median IQ (N = 58)	Above median IQ (N = 41)	All (N = 30)	ADHD/Control	IQ group differences	ADHD/Control
Gender	N female/male	16/85	7/33	9/52	4/30	12/87	6/52	6/35	3/27	ns	C_BM_ = C_AM_ = ADHD_BM_ = ADHD_AM_	ns
Age	M(SD)	10.1 (1.8)	10.1(1.8)	10.0(1.8)	10.2 (2.3)	10.5(2.0)	10.5(2.0)	10.3(2.1)	9.6 (2.3)	ns	C_BM_ = C_AM_ = ADHD_BM_ = ADHD_AM_	ns
	Range	7.0–15.7	7.1–15.1	7.0(15.7)	6.3–16.0	6.6–15.3	7.0–15.4	6.6–15.2	6.3–14.2			ns
Total IQ	M(SD)	106.0 (12.9)	93.3(7.0)	114.3(8.3)	111 (16)	101.6(16.0)	90.7(7.7)	116.9(11.7)	104 (17)	p<.05	C_BM_ = ADHD_BM_<C_AM_ = ADHD_AM_	ns
	Range	75–138	75–102	103–138	75–145	71–156	71–101	103–156	72–143			ns
Handedness	N Right/Left/Ambidextrous	90/10/1	38/2/0	52/8/1	32/2	78/16/5	42/12/4	46/4/1	25/5	ns	C_BM_ = C_AM_ = ADHD_BM_ = ADHD_AM_	ns
DISC	N ADHD-I	-	-	-	-	13	9	4	6	-	ADHD_BM_ = ADHD_AM_	-
	N ADHD-HI	-	-	-	-	21	8	13	5	-	ADHD_BM_ = ADHD_AM_	-
	N ADHD-C	-	-	-	-	65	41	24	19	-	ADHD_BM_ = ADHD_AM_	-
	N ODD/CD^a^	-	-	-	-	38	25	13	11	-	ADHD_BM_ = ADHD_AM_	-
CBCL^b^	Internalizing M(SD)	4.9(4.5)	4.5(3.8)	5.2(4.9)	6.1 (5.8)	10.1(6.4)	10.8(6.3)	9.1(6.4)	8.3 (6.6)	p<.001	C_BM_ = C_AM_<ADHD_BM_ = ADHD_AM_	ns
	Externalizing M(SD)	5.1(5.0)	4.8(5.1)	5.3(5.0)	4.8 (4.9)	18.8(10.7)	20.4(11.2)	16.6(10.7)	12.8 (8.1)	p<.001	C_BM_ = C_AM_<ADHD_BM_ = ADHD_AM_	p<.001
TRF^b^	Internalizing M(SD)	4.0(4.3)	4.5(5.5)	3.6(3.4)	2.3 (3.1)	7.6(5.8)	8.5(6.0)	6.4(5.3)	6.1 (4.8)	p<.001	C_BM_ = C_AM_<ADHD_BM_ = ADHD_AM_	p<.05
	Externalizing M(SD)	3.1(4.9)	4.8(6.6)	2.0(3.0)	2.7 (5.0)	14.2(10.7)	16.9(11.9)	10.7(7.7)	11.0 (10.5)	p<.001	C_BM_ = C_AM_ = ADHD_AM_<ADHD_BM_	p<.01
Medication^c^	% Currently using	-	-	-	-	70%	72%	66%	83%	-	ADHD_BM_ = ADHD_AM_	-
	Corrected duration M(SD)	-	-	-	-	.39(.25)	.40(.25)	.37(.27)	.36(.23)	-	ADHD_BM_ = ADHD_AM_	-

Abbreviations: ADHD, Attention-Deficit/Hyperactivity Disorder (I = inattentive type, HI = hyperactive/impulsive type, C = combined type); AM, Above Median; BM, Below Median; ODD, Oppositional Defiant Disorder; DISC-IV, Diagnostic Interview Schedule for Children-Fourth Edition; CBCL, Child Behavior Checklist; TRF, Teacher Report Form; SES, Socio-Economic Status.

a Four children that met DISC-IV criteria for ODD also met criteria for CD;

b CBCL unavailable for 2 Control_Below-median IQ_, 2 Control_Above-median IQ_, 11 ADHD_Below-median IQ_, 2 ADHD_Above-median IQ_ in structural MRI sample, for 1 Control and 3 ADHD in DTI sample; TRF unavailable for 5 Control_Below-median IQ_, 8 Control_Above-median IQ_, 12 ADHD_Below-median IQ_, 5 ADHD_Above-median IQ_ in structural MRI sample, for 1 control and 9 ADHD in DTI sample.

c Medication histories were available for 87% of ADHD_Below-median IQ_ and 79% of the ADHD_Above-median IQ_ children in the structural MRI sample and 87% of ADHD children in the DTI sample. Reported is the percentage of established use in the entire (sub)sample. Corrected duration is calculated as: duration of use in months/((age in months) – 60).

The institutional review board of the UMC Utrecht approved the study. Written informed consent was obtained from the parents of all subjects after full disclosure of the study purpose and procedure. Children provided written and/or verbal assent. The DISC-IV, parent version [61], was administered to all parents in order to confirm or disprove (controls) diagnostic status. Parents and teachers completed broad-band psychiatric screeners (Child Behavior Checklist and Teacher Report Form respectively) [62], [63]. Controls were excluded in the case of psychiatric morbidity or first-degree relatives with a history of psychiatric problems. Children with ADHD were excluded if they met DISC-IV criteria for any co-morbid disorder other than Oppositional Defiant Disorder (ODD) or Conduct Disorder (CD). In both groups, additional exclusion criteria were an IQ below 70, any major physical or neurological illnesses or the presence of metals in the body that precluded the MRI session. According to DISC-IV scores 34% of the subjects with ADHD had co-morbid ODD, and 4% CD. IQ was estimated using a four subtest short form of the Dutch version of the WISC-III (subtests Vocabulary, Block Design, Similarities and Object Assembly) [64]. Prior to the MRI-session, children aged 12 years and under participated in a practice session using a mock scanner as described previously [65]. Children over 12 years were also offered the opportunity to do a practice session.

We established the history of medication use for subjects with ADHD by reviewing the medical files. We were able to do so reliably for 83% of subjects with ADHD. Children on medication were asked not to take their medication on the day of testing. Most co-operated with this request, except for a small minority of children who were either on atomoxetine, or whose parents were not willing to abstain from medication.

### MRI Acquisition

MRI scans were acquired on a 1.5-T scanner (Philips, Best, The Netherlands). A T1-weighted three-dimensional (3D) fast field echo scan of the whole head was acquired with 130 to 150 1.5-mm contiguous coronal slices (earlier scans; 63 controls and 74 subjects with ADHD) or 160 to 180 1.2-mm contiguous coronal slices (later scans; 38 controls and 25 subjects with ADHD) (echo time [TE] 4.6 ms; repetition time [TR] 30 ms; flip angle 30°; field of view [FOV] 256 mm; in-plane voxel size 1 mm×1 mm). DTI acquisitions consisted of two transverse single shot echo planar imaging DTI scans (32 diffusion-weighted volumes with different non-collinear diffusion directions, with b-factor 1000 s/mm^2^ and 8 diffusion-unweighted volumes with b-factor 0 s/mm^2^; parallel imaging SENSE factor 2.5; flip angle 90°; 60 slices of 2.5 mm; no gap; 96×96 acquisition matrix; reconstruction matrix 128×128; FOV 240 mm; TE 88 ms; TR 9822 ms). For the analysis of the 3D-FFE scan, a mask of the intracranial space was required. For 126 subjects (60 controls, 66 subjects with ADHD) this mask was based on a T2-weighted dual echo turbo spin echo scan with 65 to 75 3.0-mm contiguous coronal slices of the whole head (echo time 1 [TE1] 14 ms; echo time 2 [TE2] 80 ms; TR 6350 ms; flip angle 90°; FOV 256 mm; in-plane voxel size 1 mm×1 mm). For 62 subjects (38 controls, 24 subjects with ADHD), the diffusion unweighted volume of the DTI scan was used to define the intracranial mask. Previous work has shown that the definition of intracranial volume is comparable using these two methods [66]. Intracranial volume masks were manually edited if necessary to ensure accuracy across scans and procedures. For 12 subjects (3 controls, 9 subjects with ADHD), the intracranial mask was traced manually on the T1-weighted image. These latter 12 masks were not used in the comparison of intracranial volume between groups.

### MRI Processing

All scans were checked for structural abnormalities by an experienced neuroradiologist. A quality check for gross movement and scanner artifacts was performed prior to processing. Only scans of good quality were used for analysis. This resulted in the data set of 214 anatomical MRI-scans in the current study. All brain scans were coded to ensure rater blindness to subject identity and diagnosis. The T1 images were first automatically placed in Talairach orientation [67] without scaling, by registering them to a model brain in Talairach orientation. The translation and rotation parameters of this registration were then applied to the images [68]. After linear registration to the T1-weighted image, the intracranial segment served as a mask for all further segmentation steps. The T1-weighted images were corrected for field inhomogeneities using the N3 algorithm [69]. An automatic image-processing pipeline was used to define the volume of total brain, cerebral and cerebellar volume, gray matter (GM), white matter (WM) of cerebrum and cerebellum, total cerebrospinal fluid (CSF) and lateral and third ventricles. The software used included updated versions of previously described histogram analysis, mathematical morphology operations, and anatomical knowledge based rules to connect all voxels of interest [70], [71]. A gray/white separation algorithm was applied accounting for effects of partial voluming [72]. The result of the separation algorithm was checked for each scan individually. Suboptimal scan quality precluded gray/white separation in 13 children (5 controls, 8 subjects with ADHD). The segments of intracranial volume, ventricles, and cerebellum were all visually checked and edited to ensure an accurate segmentation.

In order to measure cortical surface area and local cortical thickness, the binarized GM and WM segments were used as input for a custom implementation of the CLASP algorithm from the McConnell Brain Imaging Center of the Montreal Neurological Institute [73]–[75]. A 3D surface comprising 81 920 polygons and 40 962 vertices was fitted to the WM/GM interface, creating the inner surface of the cortex. The inner surface was then expanded to fit the GM/cerebrospinal fluid intersection. Total surface area of the cortex was estimated from the surface midway between the gray/white interface and the outer surface of the cortex. Cortical thickness was estimated by taking the distance between the two surfaces so that each vertex on the outer surface had a counterpart on the inner surface. For each subject, cortical thickness was calculated for every vertex and smoothed across the surface using a 20-mm (FWHM) surface-based blurring kernel [76]. This method improves the likelihood of detecting population differences, while following the curvature of the surface to preserve any anatomical boundaries. Individual surfaces were registered to the ICB-152 template [77], allowing for comparison of local cortical thickness between subjects.

### DTI processing

The two DTI scans were simultaneously realigned and corrected for possible gradient-induced distortions [78]. A robust estimation of the diffusion tensors was obtained using M-estimators to limit the influence of possible outliers [79]. FA was computed from the diffusion tensors [80]. Rigid transformations were determined to spatially align the T1 image to the diffusion-unweighted (b = 0 s/mm^2^) volume of the DTI scan using mutual information as the similarity metric. Using this transformation, the binarized cerebral WM segment from the anatomic processing pipeline described above was spatially aligned with the FA image. Mean FA was measured in this segment.

### Statistical Analyses

The primary analyses treated IQ as a dimensional construct. In order to investigate developmental trajectories, a split into separate below median and above median IQ groups was necessary (similar to Shaw and colleagues (2006)). The cutoff point for these analyses is essentially an arbitrary decision. We chose to use the whole group median IQ of 102 as the cutoff, as this allowed for subgroups of equal size and was close to the defined average IQ of 100.

Demographic data were compared between diagnostic groups using independent sample t-tests and chi-square tests, as appropriate. ANOVA was applied across diagnosis-by-IQ groups (ADHD below median, ADHD above median, control below median, control above median) with post-hoc t-tests across the four subgroups. Duration of medication use was correlated with age, therefore we calculated a corrected measure of medication use (months of use/(age in months - 60 months)), where 60 months represents the youngest age at which stimulants were prescribed in our sample. Ventricle volumes were log-transformed due to non-normality of the distribution. Age, gender, hand preference and a dummy for T1 slice thickness (1.5 versus 1.2) were used as covariates in all analyses of brain volumes and cortical thickness. All analyses of cortical thickness were corrected using false discovery rate to maintain p<.05 [81].

First, volumetric data, cerebral FA, mean cortical thickness and total cortical surface were compared between diagnostic groups using univariate GLM. An age × group term was added to the model to test for group differences in linear age effects. Differences in the relationship between volumetric measures and IQ as a continuous measure were tested by adding a main effect of IQ and an IQ × group interaction to the basic model (including the covariates and diagnostic group main effect). An alpha level of .05 was used for all univariate analyses. These analyses were repeated for the below median and above median IQ subgroups separately.

Second, to investigate differences in local cortical thickness between diagnostic groups, thickness was linearly regressed on group, age, gender, hand preference and scan slice thickness at each individual vertex. We investigated IQ effects by conducting a second regression with IQ and an IQ × group interaction added as regressors. We then investigated local differences in the developmental trajectories of cortical thickness: For each vertex, a regression analysis was carried out in the form of a locally-weighted running-line smoother [82], [83] to assess the dependence of cortical thickness on age. Fits with different degrees of freedom (df) for the age variable were calculated for each group (ADHD and controls) separately. We consecutively set df to 1 (constant), 2 (straight line), 2.2, 2.4, … (curved lines). Using the principle of parsimony, we chose the fit with the least df for each group that still described the data better than fits with lower df at alpha = 0.05 [83]. To assess whether the developmental trajectories of the groups differed from the mean trajectory, we also ran a fit for the whole group. Differences in fit across the cortical points were then investigated, applying FDR correction. These analyses were then repeated separately for below median and above median IQ subgroups.

As the density of data points was lower beyond 14 years of age and the smoothing procedure is particularly sensitive to low data densities, only cases below age 14 years were used for the cortical smoothing analyses (n_control_ = 93, n_ADHD_ = 85). Density of data points was similarly low at IQ>140, and contained only three data points for the ADHD group. These were outliers at the 1.5 IQR criterion and significantly affected the results. Therefore these cases were excluded from analyses where IQ was treated as a continuous variable (n_control_ = 102, n_ADHD_ = 96).

## Results

### Differences between diagnostic groups in brain volume, FA and cortical measures

Table 2 summarizes the results. On average, subjects with ADHD had smaller volumes of intracranium, total brain, total cerebrum, total cerebellum and smaller cortical surface area than controls (Table 2, column 8). There were no differences between diagnostic groups in the volume of lateral and third ventricles, mean cortical thickness or FA in cerebral white matter. None of the interactions between diagnostic group and age reached significance. Reductions in the volume of both cerebrum and cerebellum were primarily due to reductions in gray matter volume. When tested separately for the below median and above median IQ subgroups, these reductions in gray matter were more pronounced for the above median IQ subgroup (Table 2, column 9 and 10). Particularly, the reduction in cerebral gray matter volume was larger for subjects with ADHD and above median IQ (compared to IQ-matched controls) than for subjects with ADHD and below median IQ (compared to IQ-matched controls, 5.8% versus 3.5% respectively). The reduction in cortical surface area was comparable for both IQ subgroups (3.0%). There were no differences between diagnostic groups in local cortical thickness, nor were there any diagnostic group by age interactions or differences in developmental trajectory in local cortical thickness.

**Table 2:** Data for brain volumes and global white matter microstructure and results of the tests for main effects of group, group by age interactions and dimensional IQ effects.

Measure	Controls			ADHD			Diagnostic group effects (ADHD versus control)			Group x Age^e^	Dimensional IQ effects^f^	
	All (N = 101)	Below median IQ (N = 40)	Above median IQ (N = 61)	All (N = 99)	Below median IQ (N = 58)	Above median IQ (N = 41)	All	Below median IQ	Above median IQ			
	M(SD)	M(SD)	M(SD)	M(SD)	M(SD)	M(SD)	p	p	p	p	p_IQ_	p_IQ*group_
Intracranial Volume^a^ (ml)	1545.7(133.5)	1508.6(127.1)	1570.2(133.0)	1491.6(122.6)	1478.1(123.1)	1508.4(121.4)	**<.001**	**.038**	**.010**	.483	.075	.316
Total Brain (ml)	1393.4(117.2)	1366.4(111.6)	1411.1(118.3)	1345.9(113.5)	1335.4(114.8)	1360.7(111.3)	**.001**	**.027**	**.021**	.632	.086	.633
Total Cerebrum (ml)	1242.5(108.8)	1217.1(99.7)	1259.2(112.0)	1198.1(106.1)	1189.1(108.3)	1210.8(102.8)	**.001**	**.035**	**.020**	.486	.117	.503
Total Cerebellum (ml)	157.1(15.7)	152.0(14.0)	160.5(15.8)	151.0(12.5)	148.4(12.7)	154.7(11.3)	**.003**	.259	**.030**	.211	**.020**	.350
Lateral Ventricles (ml)^b^	9.5(5.4)	9.9(5.9)	9.2(5.2)	9.2(5.7)	9.2(5.5)	9.0(6.0)	.696	.464	.733	.436	.555	.577
Third Ventricle (ml)^b^	0.56(0.26)	0.55(0.30)	0.56(0.24)	0.54(0.29)	0.53(0.26)	0.56(0.35)	.668	.997	.750	.424	.536	.112
Cerebral Gray Matter^c^ (ml)	735.2(66.0)	714.8(59.7)	748.6(67.0)	700.5(62.7)	697.7(62.5)	704.3(63.5)	**<.001**	.070	**.001**	.419	**.023**	**.043**
Cerebral White Matter^c^ (ml)	488.8(57.0)	484.2(55.8)	491.8(58.0)	480.4(58.2)	474.9(59.8)	487.8(55.9)	.101	.123	.533	.606	.391	.420
Cerebellar Gray Matter^c^ (ml)	109.5(10.9)	106.3(11.0)	111.5(10.4)	104.6(9.5)	102.7(9.4)	107.2(9.2)	**.002**	.107	**.035**	.053	**.007**	.535
Cerebellar White Matter^c^ (ml)	46.9(8.2)	45.3(7.8)	47.9(8.4)	35.3(7.4)	44.2(6.6)	46.6(8.3)	.115	.379	.381	.630	.227	.987
Mean Cortical Thickness^c^ (mm)	3.344(0.100)	3.338(0.106)	3.348(0.097)	3.343(0.109)	3.372(0.102)	3.306(0.109)	.997	.100	.060	.108^e^	.440	**.018**
Cortical Surface Area^c^ (cm^2^)	1906.5(155.5)	1876.3(145.6)	1926.2(159.9)	1841.0(158.7)	1823.3(151.8)	1864.2(166.5)	.001	**.017**	**.055**	.729	**.018**	.877
Mean Cerebral White Matter FA^d^	0.379(0.023)	-	-	0.374(0.020)	-	-	.260	-	-	.560	.139	**.035**

Abbreviations: ADHD, Attention-Deficit/Hyperactivity Disorder; FA, Fractional Anisotropy.

Note: covariates for gender, age and slice thickness on T1 were included in all analyses (except the analysis of cerebral FA where there were no differences in slice thickness); a. n_Control_ = 98, n_ADHD_ = 90; b. raw ventricular volumes are tabulated. For analyses, these measures were log-transformed due to a deviation from normality; c. n_Control_ = 96, n_ADHD_ = 90; d. n_Control_ = 34, n_ADHD_ = 30, not split in IQ groups due to small group size; e. This column reports analyses of age effects on the whole diagnostic groups (not split by IQ). Analyses on the group with age<14 years (n_control_ = 93, n_ADHD_ = 85) showed the same pattern of results except for Mean Cortical Thickness (p = .028). Both groups showed decreasing thickness with age, but the regression line was steeper in the control group; f. This column reports analyses of IQ effects where IQ is treated as a dimensional measure, with its effects tested on the whole diagnostic groups. As these analyses were performed on continuous measures, three above median IQ outliers were excluded from structural MRI dataset for the IQ analyses (see Methods).

### Differences between diagnostic groups in the effect of IQ as a continuous measure

**Figure 1:**
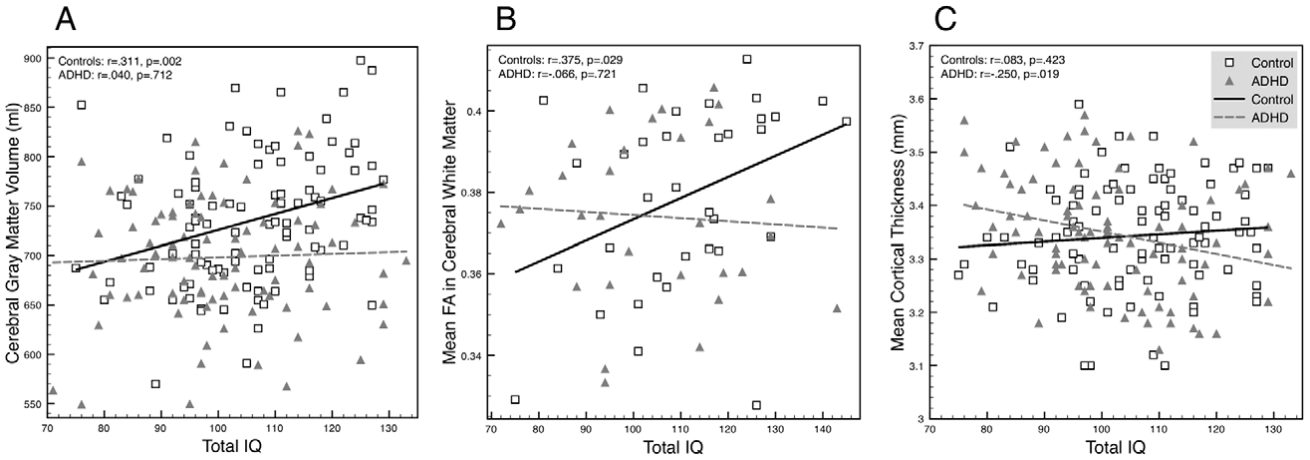
Scatterplots of measures of brain structure against IQ. Squares represent control data, triangles represent ADHD data. Linear fit lines are shown separately for the control (solid line) and ADHD groups (dashed line). For all three plots, the fits differed from one another (all p<.05; Table 2). Abbreviations: ADHD, Attention-Deficit/Hyperactivity Disorder; FA, Fractional Anisotropy.

These analyses are summarized in Table 2, final two columns. There was a main effect of IQ on the volume of total cerebellum and gray matter (both in cerebrum and cerebellum) and there were diagnostic group by IQ interactions for the volume of cerebral gray matter, FA in total cerebral white matter and mean cortical thickness (Figure 1). Specifically, for controls, IQ was positively associated with cerebral gray matter volume (r = .31, p<.01), and overall FA (r = .38, p<.05), while these correlations were not present for children with ADHD (see also Figure 1). In contrast, IQ correlated negatively with mean cortical thickness for children with ADHD (r = −.25, p<.05), but not for controls. However, there were no group by IQ interactions at any of the vertices in the vertexwise analysis of this interaction.

### Developmental trajectories of cortical thickness for IQ subgroups

**Figure 2:**
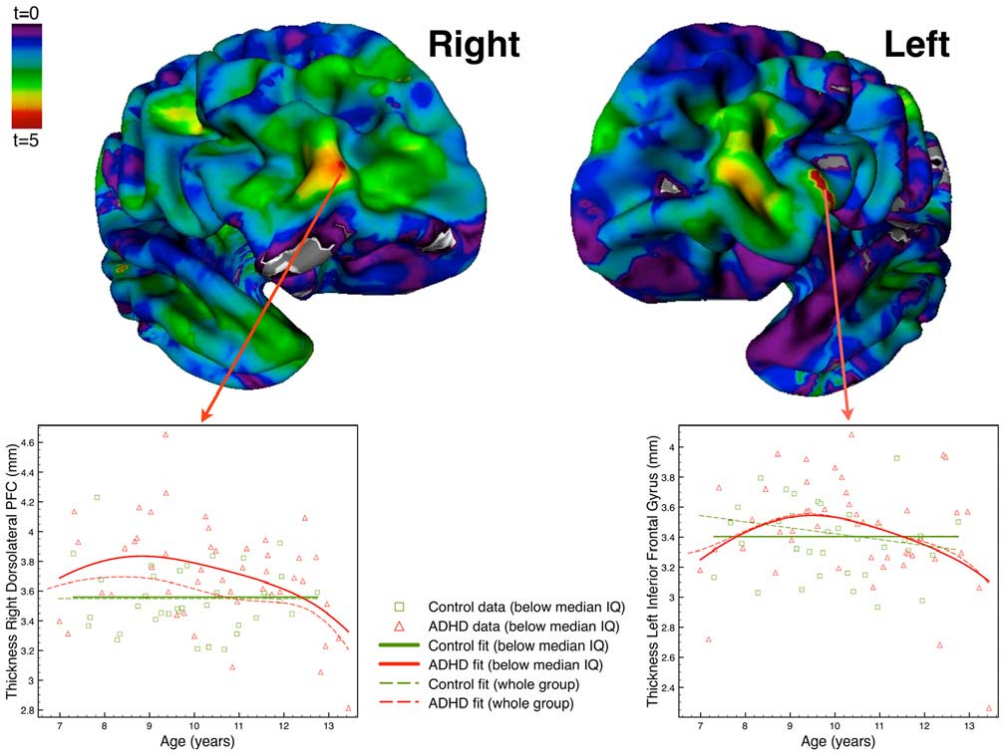
Differences in the development of cortical thickness or children with ADHD and below median IQ ADHD versus matched controls. The figure shows t-maps from the comparison of the developmental trajectories of cortical thickness between subgroups of children with ADHD and below median IQ and matched controls. Critical t-values were t = 3.69 for the right hemisphere and t = 4.27 for the left hemisphere. For the two significant prefrontal regions, scatterplots with the best fit are shown for the below median IQ data. Fits for the entire group are also shown as a reference. Abbreviations: ADHD, Attention- Deficit/Hyperactivity Disorder; PFC, prefrontal cortex.

For the subgroup with below median IQ we found differences between ADHD and control groups in the developmental trajectories of cortical thickness. Figure 2 shows the t-maps from this comparison. Children with ADHD and below median IQ had developmental trajectories that differed from those of matched controls in a number of regions including left inferior frontal gyrus (IFG) and right dorsolateral prefrontal cortex (DLPFC) (see also Figure S1).

There were no differences in the developmental pattern of cortical thickness for the above median IQ subgroup when correcting for multiple comparisons using FDR. In all regions showing differences at an exploratory threshold (p<.0001), children with ADHD and above median IQ only differed from matched controls in terms of the intercept of the developmental curve, fitting with a pattern of decreased cortical thickness that is stable across age (see also Figure S2).

## Discussion

Our results suggest that brain differences in ADHD may vary with IQ. First, we found a disruption of the typical association between measures of brain structure and IQ (Figure 1). Second, we found a pattern where ADHD combined with above median IQ was characterized by a pronounced reduction of cerebral gray matter. Analyses of the developmental trajectories of cortical thickness in above median and below median IQ subgroups suggest that this may be related to small but widespread reductions in cortical thickness that are difficult to detect in isolation but appear to be relatively stable over development. In contrast, ADHD with below median IQ was characterized less by a global reduction in gray matter but was more strongly associated with differences in the developmental trajectories of cortical thickness at specific locations on the cortex.

**Figure 3:**
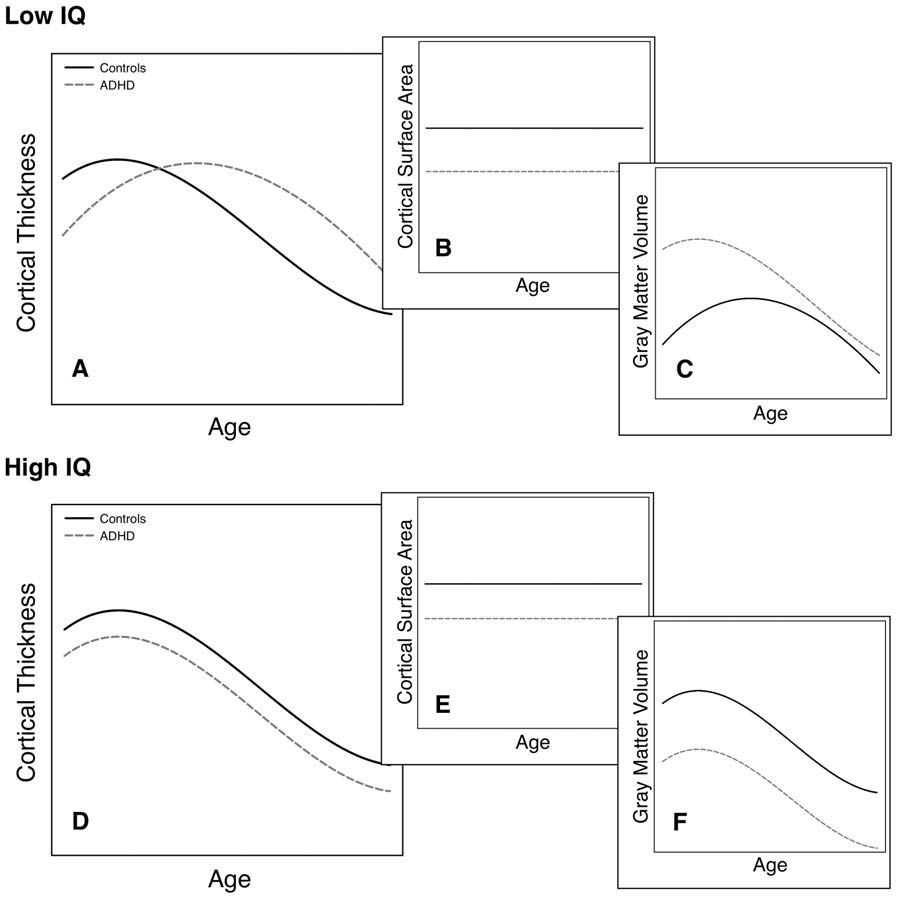
Hypothetical model of differences in cortical thickness and cerebral gray matter volume in children with ADHD and low or high IQ. 3A. In children with ADHD and low IQ, cortical peak thickness is shifted towards the right, to peak at a later age. 3B. Our results suggest a reduction in cortical surface area associated with ADHD and low IQ that is stable across age. 3C. A rightward shift in the developmental trajectory of cortical thickness combines with reduced cortical surface area (3B) to give only a minimal reduction in cerebral gray matter volume for much of the age range past the peak (using the approximation that mean cortical thickness x total cortical surface area = cortical gray matter volume, which comprises over 80% of cerebral gray matter in our data). 3D. In children with ADHD and high IQ, cortical peak volume is more similar to that of controls, resulting in more parallel trajectories with a slight difference in offset. 3E. The reduction in cortical surface area may be less pronounced in children with ADHD and high IQ than in children with ADHD and low IQ. 3F. More parallel trajectories of cortical development combined with reduced surface area (3E) will give a stable reduction in cerebral gray matter across the age range. Abbreviations: ADHD, Attention- Deficit/Hyperactivity Disorder.

For children with ADHD in the above median IQ subgroup, the developmental trajectory of cortical thickness did not differ from that of matched controls. However, these trajectories did show a developmental lag in certain areas for children with ADHD in the below median IQ subgroup. In contrast, children with ADHD and above median IQ had a marked reduction in gray matter volume, whereas children with ADHD and below median IQ did not. These findings suggest that differences in gray matter in children with ADHD and above median IQ may be more equally distributed throughout the brain and more developmentally stable than for children with ADHD and below median IQ. This model is visualized in Figure 3: In Figure 3A, the developmental trajectory of cortical thickness is shifted to the right for children with ADHD and below median IQ, leading to increased cortical thickness for children with ADHD and below median IQ at older ages. Combined with a reduction in cortical surface area that is stable across development (depicted in Figure 3B), this developmental pattern would be expected to yield attenuated or absent global gray matter deficits for a significant part of the age range (Figure 3C). In Figure 3D, the hypothetical developmental trajectory of cortical thickness for children with ADHD and above median IQ is similar to that of controls, but has a lower intercept. Combined with reduced cortical surface area in ADHD (Figure 3E), this would be expected to yield a substantial reduction in cerebral gray matter that is stable across age (Figure 3F). An alternative explanation for the combination of an overall reduction in gray matter volume and no changes in the developmental trajectories of cortical thickness in ADHD with above median IQ could be that subcortical gray matter is preferentially affected in this group, but less so in the below median IQ group.

Unlike cerebral gray matter, total cerebellar volume and cerebellar gray matter volume were positively associated with IQ for both controls and subjects with ADHD. This suggests that the genetic pathway that affects the relationship between ADHD and IQ may not mediate cerebellar volume reductions in ADHD. Nongenetic pathways may also be involved: The cerebellum is particularly vulnerable to intrauterine environmental influences and premature birth, as it starts to develop early in intrauterine life but shows a protracted developmental pattern into adulthood [84]–[87].

In the analyses contrasting the whole ADHD group to the whole control group, we found no differences in cortical thickness. Whereas we are not the first study to report such a negative finding [88], [89], a number of previous studies have shown widespread cortical thinning in ADHD [55]–[60]. We do report a pronounced reduction in cortical surface area in ADHD, previously reported in smaller samples [89], [90]. This may account for the discrepancy in our findings between reductions in cerebral gray matter and the lack of differences in cortical thickness. In addition, as Figure 3 illustrates, the developmental trajectories of cortical thickness may actually approach each other, or intersect for the below median IQ subgroup, resulting in a net absence of differences at group level.

The changes in the developmental trajectories in regions in prefrontal cortex for the below median IQ subgroup are consistent with a delayed maturation of cortical gray matter (Figure 3). However, the developmental trajectory for typically developing controls was best modeled as constant for some regions. This is likely related to the age-range included in these fits (<14 years), where a negative linear fit would have been likely with more data points at older ages. This would be consistent with the decreasing cortical thickness typically observed in the adolescent age range [25], [26], [53]. For the ADHD group with below median IQ, the best fit did show a peak in this age range, suggesting that their cortical thickness is peaking at an age when for typically developing controls it is already stable or beginning to decrease.

Studying the relationship between brain anatomy and a complex cognitive concept such as IQ is a not straightforward. Performance on tests of intellectual ability is the result of a myriad of cognitive processes, limiting the specificity of conclusions that can be drawn. However, there are also advantages to studying IQ rather than its constituent cognitive processes. The correlation between IQ and brain anatomy is one of the more consistently replicated associations between brain measures and cognition [36]. In addition, both the working definition of intelligence and its measurement are well established within both research and clinical work. As such, it may be advantageous to study a relatively general but reliable measure of cognition. Furthermore, previous studies have showed overlapping genetic effects operating on ADHD symptoms and IQ, rendering the study of the relationship between them an important step to take. That said, it will also be important to similarly relate other cognitive endophenotypes of ADHD, such as cognitive control, to measures of brain anatomy in ADHD. Specifically, familial segregation of cognitive control has been shown in ADHD that was independent from familial segregation of IQ [7], [11], suggesting that both measures carry different genetically informative variance.

Splitting the group by median IQ was necessary to perform the age-fit analyses on the cortical thickness data for different IQ levels. The choice of cut point was entirely data-driven and should not be taken to imply that ADHD groups are qualitatively different above and below this point. In addition, as a result, some of the subgroups were of modest size. However, An advantage of using a median split is that the effect of increased individual measurement error (induced by using a shortened form of the IQ test) is reduced, as this increase will have only limited effect on the group median.

One issue in any study addressing brain development in ADHD is the frequent use of medication and its effects on brain development [91]. Whereas we cannot rule out the possibility that medication use in our ADHD group affected the trajectory of brain development we feel this is unlikely to have biased the between IQ-group results, medication use was equal in the above and below median IQ groups (Table 1).

The clinical validity of ADHD across the IQ spectrum has been a source of debate. Anecdotally, many clinicians report clinically meaningful differences between subjects with ADHD at varying intellectual levels. However, empirical data on this issue is sparse. Studies that have addressed this issue have often focus on the extremes of the spectrum, i.e. intellectual disability on the one hand, or giftedness on the other [92], [93]. Especially for the latter, the pattern of clinical comorbidities, associated phenomenon and prognosis appears similar to ADHD with average IQ [92], [94], [95]. Conversely, the clinical relevance of IQ is underscored by its relationship with treatment response and functional outcome in a number of studies [14]–[19]. Whereas a neurobiological approach to this issue may have merit in the future, we would like to emphasize that all children included in the current study were rigorously assessed to show the same ADHD phenotype without comorbidity other than ODD or CD. In addition symptoms scores were also similar across the IQ subgroups. Finally, all the children included in this study were intellectually within the normal range. Therefore, a reversal of the reasoning, that a differential neurobiology across below and above median IQ in ADHD may explain clinical heterogeneity across the IQ spectrum is not warranted, based on this study alone. However, our data does suggest that in terms of neurobiology, ADHD is not independent of IQ and that a single neurobiological etiology for ADHD seems unlikely.

Our results have implications for how variance in IQ is handled in neuroimaging and cognitive studies of ADHD and reaffirm that IQ should not be used as a covariate [96], [97]. The fact that IQ has genetic overlap with ADHD in itself suggests that covarying IQ may partial out variance that is relevant to the phenotype. Our findings show that IQ is relevant to the brain phenotype of the disorder. By covarying, results are adjusted to the mean IQ value of the whole group, thus equating subjects on a measure that is genetically related to the outcome itself. Interpreting the resulting comparison is problematic from a neurobiological stance. Therefore, effects of intelligence should be actively studied rather than partialled out. This point may apply equally to other developmental disorders such as autism, where changes in IQ are an established part of the phenotype [98], and may also relate to differences in brain anatomy [99].

Finally, it is important to note that our interpretation of our results assumes neuroanatomy is the moderator between genetic variation and both IQ and ADHD. Behavioral genetic studies have implied pleiotropy: an overlapping set of genes that affect both phenotypes. Our results suggest that that this is reflected by variation in the neurobiological differences associated with ADHD as function of IQ.

In sum, we find that IQ is relevant to neuroanatomical changes in ADHD: Differences in the developmental trajectory of cortical gray matter, suggestive of delays, appear to be strongest for children with ADHD and below median IQ, whereas children with ADHD and above median IQ show widespread subtle cortical thinning that appears to be more stable over development. Our findings are based on cross-sectional data, but suggest a model for the relationship between IQ and brain anatomy in ADHD (Figure 3). Longitudinal studies will be suited to further testing this model. Nonetheless, our results are relevant to cognitive en genetic studies of ADHD in that they illustrate the importance of actively studying the effects of IQ on the phenotype.

## Supporting Information

Figure S1 **Developmental trajectories in right fusiform gyrus and calcarine cortex.** This figure shows differences in the developmental trajectories of cortical thickness for children with ADHD and below median IQ in right fusiform gyrus and calcarine cortex. The changes in trajectory for the calcarine cortex mimick the pattern found in right dorsolateral prefrontal cortex. In fusiform gyrus, the fits suggest greater cortical thickness for children with ADHD and below median IQ that is stable over development.
(TIF)

Figure S2 **Subthreshold differences in developmental trajectories of cortical thickness in children with ADHD and above median IQ.** Using FDR, there were no significant differences in the developmental trajectories of cortical thickness for the high IQ subgroup. In an exploratory analysis, we thresholded the t-maps at t(96) = 3.87, corresponding to an uncorrected p-value of .0001. Using this threshold, we did not find any significantly different vertices in the left hemisphere. In the right hemisphere, we found three clusters with changes in the developmental trajectories, in the middle occipital gyrus, in the temporal pole and in insular cortex. For each cluster, the difference in the trajectory was mostly attributable to a difference in intercept, consistent with a stable decrease in cortical thickness for children with ADHD and above median IQ. This is consistent with the hypotheses presented in the main paper (Figure 3).
(TIF)
