# Supplementary figures and images for: Differential Brain Development with Low and High IQ in Attention-Deficit/Hyperactivity Disorder
Source: PLoS One. 2012 Apr 20;7(4):e35770. doi: 10.1371/journal.pone.0035770 (PMC3335015; doi:10.1371/journal.pone.0035770)

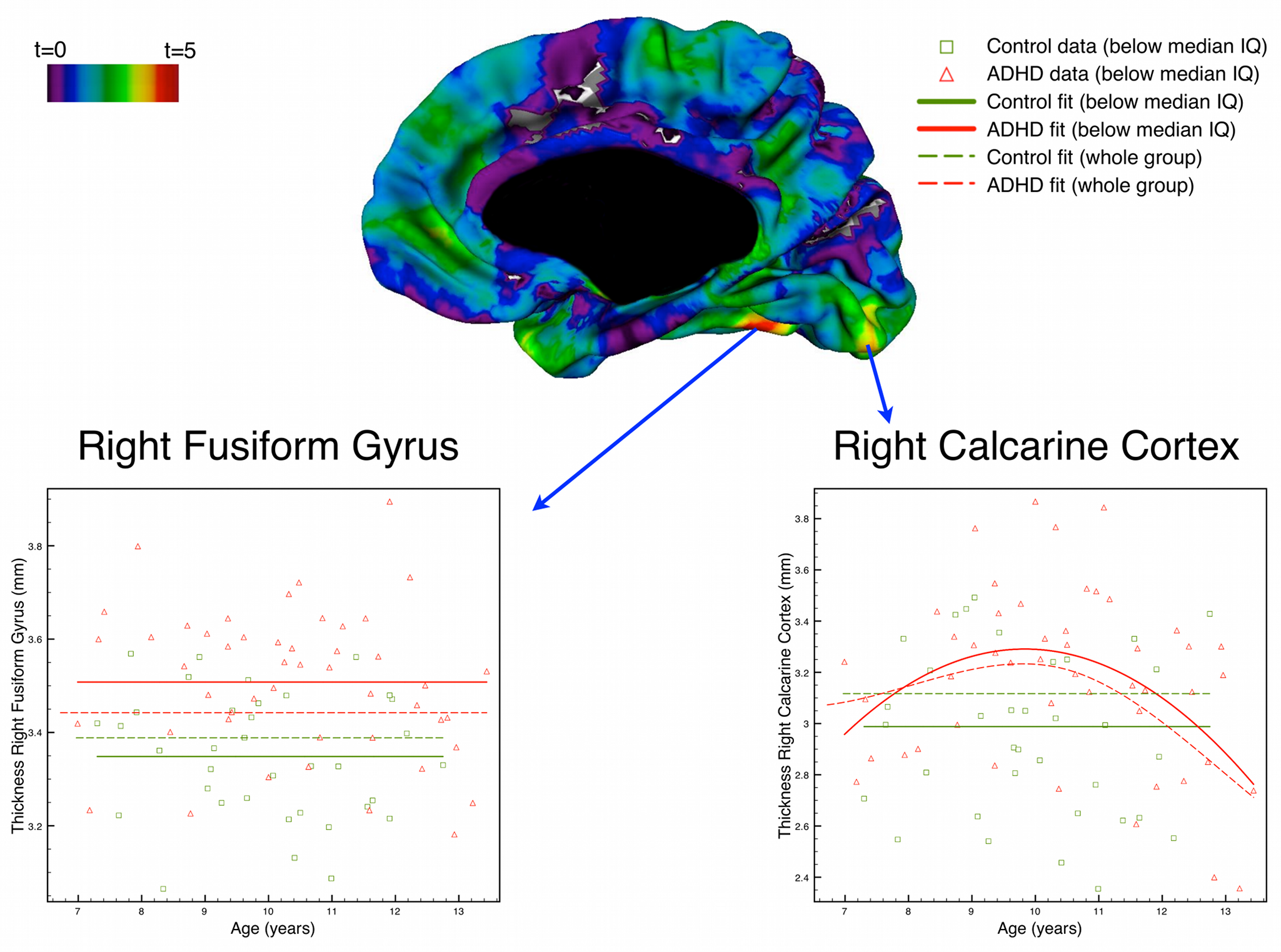

Supplement: Figure S1 — Developmental trajectories in right fusiform gyrus and calcarine cortex. This figure shows differences in the developmental trajectories of cortical thickness for children with ADHD and below median IQ in right fusiform gyrus and calcarine cortex. The changes in trajectory for the calcarine cortex mimick the pattern found in right dorsolateral prefrontal cortex. In fusiform gyrus, the fits suggest greater cortical thickness for children with ADHD and below median IQ that is stable over development. (TIF) [file pone.0035770.s001.tif]

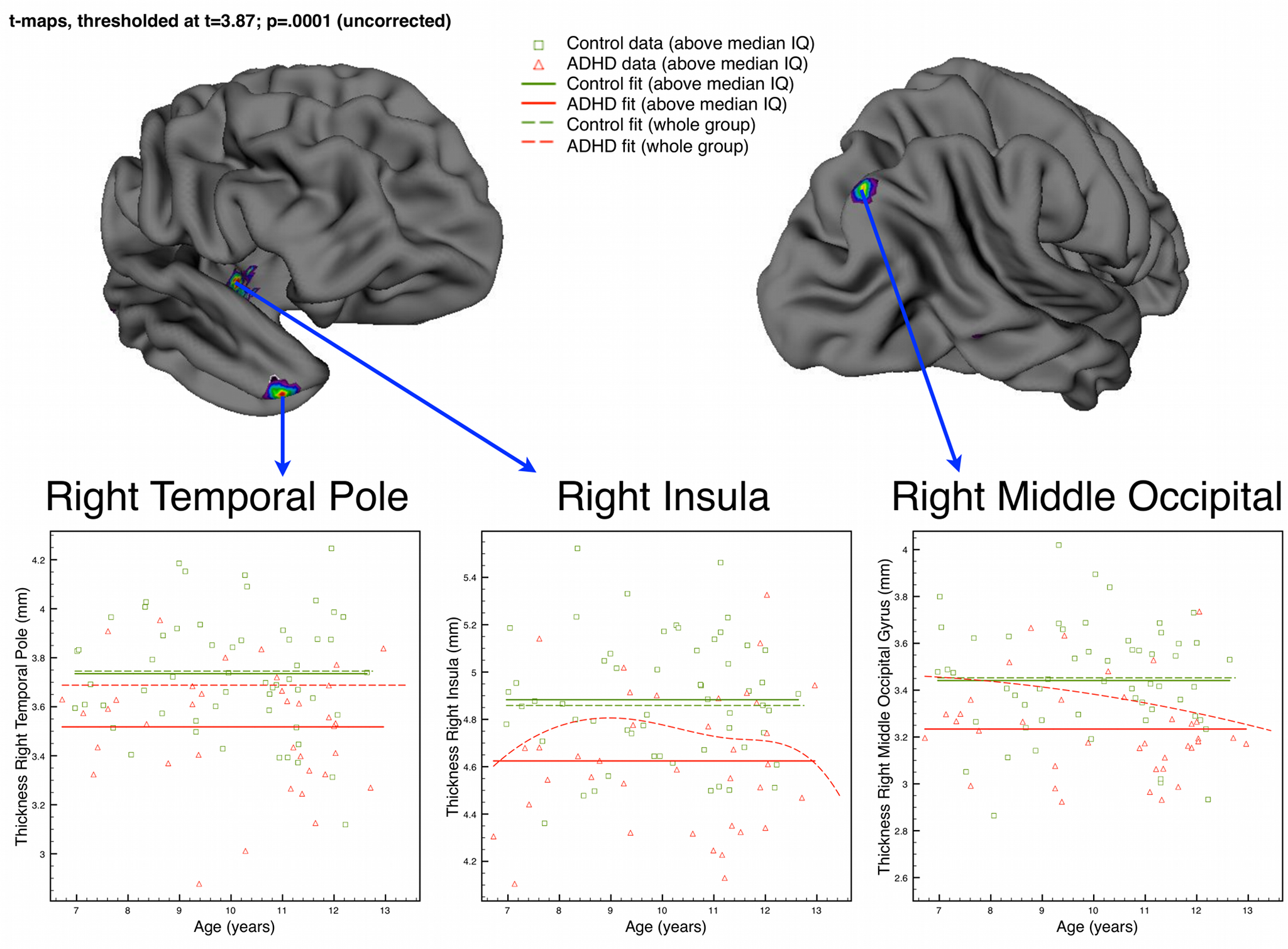

Supplement: Figure S2 — Subthreshold differences in developmental trajectories of cortical thickness in children with ADHD and above median IQ. Using FDR, there were no significant differences in the developmental trajectories of cortical thickness for the high IQ subgroup. In an exploratory analysis, we thresholded the t-maps at t(96) = 3.87, corresponding to an uncorrected p-value of .0001. Using this threshold, we did not find any significantly different vertices in the left hemisphere. In the right hemisphere, we found three clusters with changes in the developmental trajectories, in the middle occipital gyrus, in the temporal pole and in insular cortex. For each cluster, the difference in the trajectory was mostly attributable to a difference in intercept, consistent with a stable decrease in cortical thickness for children with ADHD and above median IQ. This is consistent with the hypotheses presented in the main paper (Figure 3). (TIF) [file pone.0035770.s002.tif]
